# Characterizing the Bioactive Ingredients in Sesame Oil Affected by Multiple Roasting Methods

**DOI:** 10.3390/foods11152261

**Published:** 2022-07-28

**Authors:** Hossam S. El-Beltagi, Rabab W. Maraei, Abeer E. El-Ansary, Adel A. Rezk, Abdallah Tageldein Mansour, Amina A. Aly

**Affiliations:** 1 Agricultural Biotechnology Department, College of Agriculture and Food Sciences, King Faisal University, P.O. Box 400, Al-Ahsa 31982, Saudi Arabia; arazk@kfu.edu.sa; 2 Biochemistry Department, Faculty of Agriculture, Cairo University, Giza 12613, Egypt; abeeransary@yahoo.com; 3 Natural Products Department, National Center for Radiation Research and Technology, Egyptian Atomic Energy Authority, Cairo 11787, Egypt; alrahman_27israa@yahoo.com; 4 Plant Pathology Research Institute, Agricultural Research Centre, Giza 12619, Egypt; 5 Animal and Fish Production Department, College of Agricultural and Food Sciences, King Faisal University, P.O. Box 420, Al-Ahsa 31982, Saudi Arabia; amansour@kfu.edu.sa; 6 Fish and Animal Production Department, Faculty of Agriculture (Saba Basha), Alexandria University, Alexandria 21531, Egypt

**Keywords:** fatty acid profiles, FTIR, physicochemical characteristics, roasting methods, *Sesamum indicum*

## Abstract

Roasting is an important step in sesame (*Sesamum indicum* L.) processing. The current research was undertaken to evaluate the oil content, fatty acid (FA) profiles, and physicochemical characteristics of oil recovered from sesame roasted by different methods (cooker oven, stovetop pan, microwave, and electric frying pan). Roasting sesame seeds changed their oil content according to the roasting method used, with content ranging from 49.83% in control to 59.85% in the roasting by microwave. In oils recovered from raw or roasted seeds, seven fatty acids were obtained through gas chromatography. Changes in the fatty acid profiles occurred in all the treatments, and the total unsaturated fatty acid content was higher than that of saturated fatty acids. The obtained peroxide number of sesame oils was inside the rate of 3.90 meq/kg oil for microwave treatment versus 1.59 meq/kg oil for unroasted. The highest acid value was with the stovetop pan treatment at 3.78 mg/g, followed by the microwave treatment at 3.24 mg/g; the oven treatment gave the lowest value at 1.66 mg/g. The lowest iodine value was observed with the electric frying pan treatment (102.30/100 g oil), and phytosterols were most abundant with the microwave treatment. Moreover, the phenolic and flavonoid contents and antioxidant activity were the highest with the microwave roasting. The FTIR spectrum illustrated slight differences in peaks intensity (1738, 1454, 1151, 710 cm^−1^) between the roasting methods used. The finding of the current investigation of roasting methods was that the fatty acid profiles were across methods. As is clear from the obtained results, the microwave roasting treatment is the favoured roasting method for the healthiest sesame seed oil contents. Sesame seeds are considered a significant and abundant resource with numerous beneficial nutrients that positively affect human health.

## 1. Introduction

Sesame (*Sesamum indicum* L.) is an oilseed crop of the Pedaliaceae family that is grown in several developing nations but that is known for its high labor demands for cultivation and harvesting [1]. The average yields of sesame seeds reported in a Food and Agricultural Organization report (FAO, 2018) ranged from 256 to 1400 kg/ha. The three countries producing the highest yield were Mainland China), Nigeria, and United Republic of Tanzania at 1400, 1100, and 1074 kg/ha, respectively [2]. Among the oilseed crops, sesame ranks eighth in terms of oil production globally [3]. According to the FAO, the average global sesame seed oil production in 2014 was 1.63 million tons. Sesame is a significant annual oil seed crop used in the production of cooking oils, flour, and animal feed. The sesame seed oil contains saturated fatty acids, unsaturated fatty acids, and protein as well as slight amounts of different nutrients like lignans such as sesamin, sesamol, and sesamolin as well as tocopherol [4,5,6]. Sesame is also rich in minerals and vitamins such as iron, magnesium, copper, calcium, phytosterol, and vitamins B_1_ (thiamine) and E (tocopherols) [7]. The plant sterols in the oils are associated with reducing the absorption of cholesterol from the diet, leading to declining LDL cholesterol levels and reduced risk of cardiovascular illness [8]. Sesame has a variety of food functions, for example in crackers, bread, cookies, biscuits, hamburger, sauces, and desserts as well as salad dressing. Sesame oils, which comprise about half of the seed, are utilized in different processed items like cooking oils, salad oils, soaps, lubricant, creams, pesticides, and several pharmaceuticals [9,10].

Owing to the widespread understanding of the health benefits of sesame seeds, significant research on its applications has taken place in recent years [11]. Sesame has also great potential for enhancing the shelf life and longevity of other oils with its bioactive components [12]. Prior to oil recovery and use, sesame seeds are exposed to some pre-treatment consisting of dehulling, wetting, roasting, steaming, and heating to increase the oil extracted and thereby the value [13]. Among these pre-treatments, roasting is the most commonly utilized; it causes physical alterations in the seed properties that can have a significant impact on the oil component contents [12,14]. Roasting amplifies the sensory, nutritional, and health properties of sesame oil [13]. Furthermore, roasting improves the oxidation stability or antioxidant activity of sesame oil because it liberates numerous bioactive substances like sesamol, sesamin, sesamolin, and phenolic compounds apart from tocopherols [15]. In addition to these bioactive components, roasting encourages the development of Maillard reaction products (MRPs), which also show antioxidant properties and protect the sesame oil from oxidation [16]. Notably however, due to the Maillard reaction, poor roasting of sesame seeds may result in the creation of unsafe pollutants like furans and acrylamides, as well as causing vitamin decay and activating protein aggregation, breakdown, and unfolding [16]. Roasting is a classic food process concerning thermal drying in which warm air wraps the meal, roasting it evenly on all sides [17]. Roasting can improve the digestibility and tastiness of meals while improving the bioavailability of constituents via physicochemical and structural changes. Roasting also improves colour, flavour, active taste substances, and antioxidants [18]. Roasting additionally can remove or decrease natural poisons and inhibit enzymes and microbial load [18]. The roasting period may differ depending on the wanted features of the ending products [19]. The roasting processing is divided into three stages: drying, roasting, and colour/taste creation [20]. Roasting can cause alterations in the metabolite contents (phytosterols, vitamins, fatty acids) of oils [21]. The time required for microwave processing is greatly shortened in the foods. Those interactions directly produce molecular resistance and excitation [22]. One of the main processes that occur in roasting fat-containing foods is oxidation, oxidation products can interact with other food component to form both desirable and undesirable compounds [23]. The degree of fat oxidation and saturation is determined by the estimated oxidizability (COX) or saturation fat (S/P) indices. Fats with high S/P proportions have more favourable fatty acid stability than fats with a low S/P [24]. The proportion between monounsaturated- (OL) and poly-unsaturated (LA + ALA) acids is a good predictor of oil value [25].

The goals of the current study were to conduct thermogravimetric analysis of the oil properties along with the fatty acid profiles, bioactive ingredient (tocopherols, sterols), chlorophyll, and carotenoid contents in sesame oils obtained from seeds roasted by different method (cooker oven, stovetop pan, microwave, or electric frying pan).

## 2. Materials and Methods

### 2.1. Roasting Process

The seed materials used for this study fresh white sesame (*Sesamum indicum* L.) seeds collected from a local market in Cairo, Egypt. The raw sesame seeds were divided into 5 sections of 200 g each, one unroasted and the other 4 parts exposed to the different roasting methods (cooker oven, stovetop pan, microwave, or electric frying pan). Roasting was stopped when the seeds reached the desired color. All experiments were performed in three replicates.

Specifications of the devices used in roasting:

Cooker oven

The sesame seeds were roasted in a ceramic pan in a conventional hot-air cooker oven (Techno-gas, M-GC Mystro cooker with fan and 5 burners, silver, Egypt) at 180 °C.

Stovetop pan

The sesame seeds were roasted in a ceramic pan on the stovetop (Techno-gas, M-GC Mystro cooker with fan and 5 burners, silver, Egypt) at 180 °C.

Microwave

Sesame seeds were placed in a thin layer in a Pyrex plate (25 cm diameter) and heated in a microwave oven (Black and Decker, 230–240 V, 50 Hz, 1400–1450 W, China).

Electric frying pan

Sesame seeds were roasted using an air fryer (Philips XL, black, 94–60 Hz) at 180 °C with constant stirring. After the roasting, the seeds were left to cool to room temperature for one h in desiccators.

The crude oils were recovered from both the unroasted and roasted sesame seeds utilizing a Soxhlet system [26]. Briefly, 25 g of each grounded sample were used for oil extraction with 250 mL of petroleum ether at 60 °C for about 6 h. Solvent was then evaporated via rotary evaporator (rotary evaporator RE-501, 60 W, evaporation rate L/h, flask rotation speed 150 rpm, Shanghai, China) and the obtained oils were reserved in sealed glass bottles at −20 °C until they were needed. This was repeated until all the different assays were completed.

The percent oil yield was calculated using the following formula:

Percent of oil = W_2_/W_1_ × 100

where W_2_ is the weight of the extracted oil and W_1_ is the weight of the sesame seed sample.

### 2.2. Chemical Properties of Oil

#### 2.2.1. Peroxide Value (PV)

Standard procedures were used to estimate PV asmeq O_2_ kg^−1^ oil [27]. Into a 250 mL Erlenmeyer flask, one gram of the oil sample, 1.0 g of potassium iodide, and 20 mL of solvent mixture (glacial acetic acid/chloroform, 3/2 by volume) were added, and the mixture was heated and allowed to boil for one minute. The hot solution was then poured into a flask containing 20 mL of 5% potassium iodide. Thereafter, 3 drops of starch solution were added to the mixture and titrated with 0.025 N standardized Na_2_S_2_O_3_. The peroxide value was determined following the equation:

Peroxide value = S × N × 100/W

where, S = vol. in mL of Na_2_S_2_O_3_, N = normality of Na_2_S_2_O_3_, and W = weight of oil sample (g).

#### 2.2.2. Acid Value (AV)

To measure AV as oleic acid % in mg KOH g^−1^ oil [28], each oil sample (2.0 g) was weighed into a conical flask. Afterwards 5 mL of chloroform was added, and a mixture of 25 mL diethyl ether and ethanol 1:1 (*v/v*) was also added. A few drops of phenolphthalein indicator were also added, and the mixture was titrated against 0.1M KOH. The end point was noted when the pink colour appeared and persisted for 30 s. The acid value was calculated as:

Acid Value = a − b × M × 56.1/W × 100

where a = vol. in mL of sample, b = vol. in mL of blank, M = morality of KOH, and W = weight of oil sample (g)

#### 2.2.3. Iodine Value (IV)

To measure the IV in g I_2_ 100 g^−1^ [29], an oil sample (0.30 g) was dissolved in 10 mL of chloroform in 100 mL glass stoppered flask. After that, 25 mL of Wijj’s solution (iodine monochloride) was added, and the flask stood in a dark place for 30 min. Then, 20 mL of 10% KI was then added, and the mixture was titrated against 0.1M sodium thiosulphate with a few drops of starch as indicator. A blank titration was also carried out. The iodine value was calculated using the following equation:

Iodine value = (a − b) × 1.269/W × 100

where a = sample titre value, b = blank titre value, and W = weight of sample used (g).

#### 2.2.4. Saponification Value (SV)

The measure the SV as mg KOH g^−1^ sesame oil [28], each oil sample (2.0) was weighed in a conical flask and dissolved with 5 mL of chloroform; then, 25 mL of 0.5 M alcoholic KOH was added. The flask was corked, and the mixture was refluxed for 30 min. The mixture was then transferred into a conical flask, a few drops of phenolphthalein indicator was added, and it was titrated against 0.5 M HCl until the pink colour disappeared, indicating the end point. The saponification value was calculated thus:

Saponification value = (a − b) × M × 56.1/W × 100

where a = sample titre value, b = blank titre value, M = molarity of the HCl, and 56.1 = molecular weight of KOH. Ester value (EV) was evaluated as saponification value–acid value.

### 2.3. Fatty Acids Profile

Fatty acid methyl esters (FAME) were made in accordance with IUPAC [30]. A Shimadzu GC-2010 chromatograph with fused capillary DB-23 fused-silica column (0.25 mm i.d., 60 m, 0.25 μm film thickness, Agilent J&W, Santa Clara, CA, USA) was used for GC analysis. Helium was used as the carrier gas at flow rate of 0.70 mL/min. Column temperature was adjusted to isothermal at 190 °C for 95 min, where injector and detector temperatures were 230 and 240 °C, respectively. The FAME peak areas were discovered via comparing retention time to fatty acid reference standards. These standards included capric acid (C10:0), lauric acid (C12:0), myristic acid (C14:0), palmitic acid (C16:0), stearic acid (C18:0), arachidic acid (C20:0), lignoceric acid (C24:0) palmitoleic acid (C16:1), oleic acid (C18:1), linoleic acid (C18:2), linolenic acid (C18:3), and erucic acid (C22:1).

### 2.4. Calculated Oxidizability Value (Cox)

Based on the quantities of unsaturated fatty acids (USFAs), the oxidative stability of each various roasting method was estimated [31]:

Oxidizability = [1 (18:1%) + 10.3 (18:2%) + 21.6 (18:3%)]/100.

### 2.5. Oleic Desaturation Ratio (ODR) or Linoleic Desaturation Ratio (LDR) Values

Pleines and Friedt [32] calculated the ORD or LDR to evaluate the efficiency of desaturation from oleic to linoleic (ODR) acid or from linoleic to linolenic (LDR) acid within the desaturation process. They were calculated as follows:

ODR = %C18:2 + %C18: 3/%C18:1 + %C18:2 + %C18:3

LDR = %C18:3/%C18:2 + %C18:3

### 2.6. Chlorophylls and Carotenoids Contents

The chlorophyll and carotenoid concentrations in the oil samples were assessedin cyclohexane following the protocol of Chtourou et al. [33]. The absorbance of each sample was observed at 670 nm for chlorophyll and 470 nm for carotenoid using aspectrophotometer (Jasco model V 530, Tokyo, Japan). Their concentrations were calculated using formula (1) for chlorophylls and formula (2) or carotenoids:

Chlorophyll (mg/kg) = (A670 × 106)/(613 × 100 × d)(1)

Carotenoids (mg/kg) = (A470 × 106)/(2000 × 100 × d)(2)

where A, absorbance of the oil at the respective wavelength and d, thickness of the cuvette (1 cm). The chlorophyll and carotenoid contents are stated as mg per g of oil.

### 2.7. Conjugated Dienes and Trienes

Approximately 20 mg of sample was diluted in 10 mL n-hexane or carefully mixed. Absorption was determined in the UV range with aspectrophotometer (Jasco V530, Tokyo, Japan) at 233 and 268 nm for conjugated diene and triene, respectively, following the IUPAC method II D.23 [30].

The CD and CT values were calculated using Equations (3) and (4), respectively:

CCD = Abs232/C × L(3)

CCT = Abs268/C × L(4)

where C is content of oils in g/100 mL, L is length of cuvette, CCD and CCT are conjugated dienes (CD) or conjugated trienes, and (CT) content (%) of oil samples.

### 2.8. Phytosterols and Tocopherols

#### 2.8.1. Sample Preparation for HPLC Analysis

For the lipids extracted by Soxhlet methodology, 0.2 g fat was dissolved in 20% alcoholic potassium hydroxide (25 mL), and the solution was refluxed at 85 °C for 4 h to ensure complete hydrolysis. The non-saponified portion was extracted three times with diethyl ether. The combined ether layer was collected and washed with distilled water to remove the saponified portion and then dried over anhydrous sodium sulphate. The solvent was then evaporated to dryness. The residue was dissolved in HPLC-grade methanol and filtered through a 0.2 µm PTFE filter and used for HPLC analysis.

#### 2.8.2. HPLC Conditions

The chromatographic separation was performed on an Agilent 1260 infinity HPLC series (Agilent^®^, Santa Clara, CA, USA) outfitted with quaternary pump. The samples were injected onto a column, Hyper Clone TM BDS C18, 130 Å 100 mm × 4.6 mm (Phenomenex^®^, Torrance, CA, USA), and the injection volume was 20 μL. The separation was achieved using isocratic elution with acetonitrile: methanol (70:30). The flow rate was 1.0 mL min^−1^, and the column was maintained at 35 °C. For tocopherols, the detection was performed with a fluorescence detector (excitation wavelength = 230 nm and emission wavelength = 460 nm), while sterols were detected with a VWD detector set at 205 nm. The tocopherols and phytosterols were identified by comparison with authentic standard retention times. The sterol and tocopherol contents were expressed as mg per kg oil.

### 2.9. Phenol Contents

Phenol amounts in oils obtained from raw and roasted samples were determined using theFolin–Ciocalteu technique with agallic acid standard curve [34]. The oil (0.5 g) was extracted in methanol (5.0 mL) by inversion for 10 min. The methanolic extract (1.0 mL) was mixed with Folin–Ciocalteu reagent (1.5 mL). After 3 min, 20% Na_2_CO_3_ solution (2.5 mL) was added, and the mixture was diluted up to 10.0 mL with distilled water. The solution was allowed to stay at room temperature. After 45 min, the mixture was centrifuged, and absorbance was calculated utilizing a spectrophotometer (Jasco V530, Tokyo, Japan) at 725 nm. Values are given as milligram gallic acid per gram of oil.

### 2.10. Total Flavonoid Content

Aluminium colorimetry was utilized to evaluate the flavonoid contents. Absorbance rate was estimated at 510 nm by spectrophotometer (JascoV530, Tokyo, Japan) [35]. Briefly, 0.5 mL methanolic extract was mixed with 2.5 mL of distilled water in a test tube, followed by adding 0.15 mL of a 5% NaNO_2_ solution. The mixture was left at room temperature for 6 min. Then, 0.30 mL of a 10% AlCl_3_·6H_2_O solution was added and allowed to stand for another 5 min before 1.0 mL of 1.0 M NaOH was added. The mixture was increased to 5.0 mL with distilled water and mixed well. The absorbance was measured at 510 nm. The obtained result was stated as milligram of quercetin per gram of recovered oil.

### 2.11. Antioxidant Activity

Radical scavenger action of treatments against the 2,2-diphenyl-1-picryl hydrazyl (DPPH) radical was established using the technique described by Gulluce et al. [36] with some modifications. The oil sample (0.1 g) was dissolved in n-hexane, the volume was increased to 1.0 mL, and 40 μM DPPH solution prepared in n-hexane (2.9 mL) was added to the sample. The solution was allowed to stand for 30 min at room temperature, and the absorbance was recorded at 517 nm. The solutions were prepared preferably in hexane because the methanolic solution of DPPH causes turbidity in the reaction mixture and thus interferes with the results. The radical scavenging activity was calculated using the following formula:

% DPPH scavenging activity = [AC − AS/AC] × 100

where AC is the absorbance of the control reaction (containing all reagents except the sample) and AS is the absorbance when the sample extract is added.

### 2.12. Fourier Transforms Infrared (FTIR) Spectroscopy

The FTIR spectra of the oil extracted from unroasted and roasted sesame seeds were obtained using anFTIR spectrometer (Bruker, Unicom, Flensburg, Germany) at room temperature, and spectra were acquired in the range 4000–500 cm^−1^.

### 2.13. Thermogravimetric Analysis (TGA)

The TGA measurement of oil recovered from the unroasted as well as roasted sesame seeds was performed with the Shimadzu-50 device (Kyoto, Japan) at 10 °C/minute under a nitrogen flow rate of 20 mL/min with thermal range of 10 °C/minute for nitrogen flowing (20 mL/min) from room temperature to 600 °C.

### 2.14. Statistical Analysis

Three replicates were used in a randomized full block design, and the results were presented as means ± standard deviations. Statistical analysis was performed via one-way ANOVA, and the divergences in means were calculated utilizing Duncan’s multiple rate tests [37] with *p* ≤ 0.05. The statistical software used was CoStat (version 6.311) statistical CoHort software (Berkeley, CA, USA).

## 3. Results and Discussion

The oil content recovered from sesame seeds roasted by different roasting methods were evaluated and compared with that of unroasted. Data in Table 1 showed that total lipids in sesame oil increased with roasting: the oil content in the control was 49.83%, followed by roasting in a microwave treatment (59.85%) and in an electric frying pan (59.75%). In short, the findings showed that roasting sesame seeds increased oil yield, and thus, it might be utilized as a pre-treatment approach prior to oil recovering to enhance oil content. The current results are in agreement with Hojjati et al. [38], who reported that the oil content of wild almonds increased with microwave roasting as microwave heating evaporate water from the interior of the almonds’ microstructure, increasing the pressure inside them, and causing the material disintegration by rupturing cell membranes and increasing porosity, which leads to improve oil recovery efficiency by enabling oil passage.

Microwave roasting has been shown to improve rape seed oil press efficiency [39] as well as peanut oil [40]. The lesser oil yield of unroasted oil seeds was attributable to the intactness of cell wall breakage through pressing, whereas after roasting the cell walls undertakes stable alters in porosity and allowing the liberate of oil and that increases the oil recovery effectiveness [41]. Additionally, the protein and oil content increase due to water loss from heat processing [42]. Indeed, a previous study [43] observed variations in fat content and slight increases in trans fatty acid (TFA) contents upon heating in almonds as well as pistachios.

### 3.1. Peroxide Value (PV)

Peroxide value is utilized to measure oil value via evaluating the development of primary oxidation products formed because of the oxidative deterioration of oils [44]. The sesame oil peroxide values were presented in Table 1. Regarding to the different roasting treatments, the PV of sesame oil was under 1.59 for unroasted and increased to 3.90 (meq/kg) with microwave roasting. The increased PV might be attributable to the acceleration of dry heat in the presence of air leading to the breakdown and decomposition of fatty acids to form peroxide compounds. The peroxide value in the different treatments was less than 5 meq/kg, an acceptable level for oils according to the FAO/WHO [45].

A comparable effect was obtained by Potočnik et al. [46]. The detected results are due to the attack of free radicals on unsaturated fatty acids, resulting in the build-up of hydroperoxides, which can improve peroxide value in early phases or reduce peroxide value at high roasting temp [47]. Meanwhile, previous study found that with roasting, the peroxide value was significantly higher, and the authors suggested that this may be an effect of the thermal oxidation of USFA in sesame oil [11]. On the other hand, microwave considerably enhanced the value of fat in seed [48], which resulted in a considerably lower peroxide value. Nevertheless, microwave heating is linked to the creation of oxygenated compounds and fatty acid conjugates, lowering oil constancy and increasing oil rancidity.

### 3.2. Iodine Value (IV)

A high IV may be considered to indicate high USFA content. Here, IV was 105.10/100 g for unroasted and decreased for all roasting methods reaching its minimum value in the electric frying pan (102.30/100 g). This reveals that heat caused the breakdown of the double bonds in the sesame oil, and the polymerization, oxidation, or breaking of long-chain fatty acids may have reduced the amount of un-saturation positions, resulting in these decreases [49]. Oils with high iodine value contain more double bonds than oils with lower iodine value, so oils with high iodine valueshow less oxidative stability [50]. The results for the current study align with those from Makinde et al. [51] for roasted sesame oil.

### 3.3. Acid Value (AV)

The highest acid value was found with the stovetop pan treatment at 3.78 mg/g, followed by the microwave treatment at 3.24 mg/g; the unroasted gave the lowest value (1.03 mg/g). Previously, sesame oil acid content increased with increasing roasting temperature [11]. This rise might be attributed to the oil’s thermal degradation as well as oxidation, of which generates many volatile substances like alcohols, aldehydes, alkanes, carboxylic acids. Additionally, many of these volatile chemicals participate in Maillard reaction or generate fragrance compounds [11], and a high number usually indicates that the sesame seeds were heavily enzymatically hydrolysed through harvesting, handling, or oil extraction [52].

### 3.4. Saponification Value (SV)

The SV indicates the free or esterified acids found in fats and fatty acids, measured as mg of potassium hydroxide necessary for neutralizing free acids and saponifying esters in one gram of oils [53]. SV in the present study ranged from 189.8 mg/g for the unroasted seeds to 195.3 mg/g with oven roasting. The obtained data are consistent with Rizki et al. [54], who found higher SV for roasted than for unroasted sesame seed samples. Furthermore, the current findings are within the range of the Codex Standard [55] for sesame oil saponification of between 186 and 195 mg KOH/g.

### 3.5. Ester Value (EV)

EV indicates the acidity of sesame oil measured as the ester index. In this study, it was noted that unroasted seed oil had a high ester content, confirming its high acidity (Table 1). Ester value reached its maximum with oven roasting (193.7 mg/g), compared with unroasted (188.8 mg/g). The high SVs in the oil point to fatty acids with low molecular weights and short chains, and low SVs indicate fewer ester bonds is than normal [56]. Yen [57] reported that sesame oils prepared at roasting temperatures between 180 and 220 °C have no obvious differences in properties, such as acid value, saponification value, and refractive index.

### 3.6. Fatty Acid Profiles of Sesame Oil

The fatty acid (FAs) profile is an important marker of the nutrition quality of oil; sesame seed oil belongs to the oleic–linoleic acid group [58]. The fatty acid profiles of sesame seeds roasted with different methods are shown in Table 2. Oleic and linoleic acids accounted for more than 84% of all the fatty acids in the sesame seed oils obtained by different roasting methods.

The proportion of saturated fatty acids (SFAs) was highest (14.56%) in the oil recovered from oven roasted seeds, attributed to the high palmitic acid content, 8.53%. The most unsaturated fatty acids (UFAs) were in the oil recovered from the microwave roasted seeds (86.69%), attributed to the high linoleic acid content (C18:2). The SFA/USFA ratios for the different roasting methods ranged from 0.1620 to 0.1688; the variations according to roasting method were slight and not significant (Table 2), with the highest ratio for the sesame seed oil recovered from oven-roasted seeds (0.1688%). The contents of these fatty acids in the different examined oils were within the Codex range [59]. Meanwhile, Lin et al. [60] found that almond kernels heated at 180 °C for 20 min had the most USFAs and SFAs. Furthermore, Hama [61] noticed various changes in fatty acids through roasting, with some increasing while others decreased or stay unchanged. The fatty acid profile of the sesame seed oil was not altered by roasting [10]. The differences among these investigations might be because of variations in sesame seed genotypes and roasting methods. Goszkiewicz et al. [48] found that roasting seeds in a microwave guaranteed significant defence against hydrolytic rancidity and decreased the primary oxidation compounds, which had significant impacts on the shelf life of the product. The sesame seed oils recovered from unroasted and roasted seeds in the current investigation contain large amounts of UFAs (>84%) like oleic and linoleic acids as well as significant amounts of palmitic acid, which demonstrate high nutritional qualities. Unsaturated fatty acids are recognized for their pre-emptive effects against distinct cardiovascular diseases; for instance, linoleic acid is identified to lower serum cholesterol or LDL, oleic acid may raise HDL, and palmitic acid might have hypercholesterolaemic action after esterification at β-site [42,62].

### 3.7. Ratio of Palmitic (P), Stearic (S), Oleic (O) over Linoleic Acid and SFAs/USFAs

The palmitic/linoleic, stearic/linoleic, and oleic/linoleic ratios of sesame seeds roasted by different methods in this research were comparable with those reported. The obtained fatty acids and their ratios showed little change according to roasting method (Table 3).

A favourable saturated fatty acid to unsaturated fatty acid proportion may be considered an important guide for measuring eatable oil value, and the beneficial ratios of palmitic, stearic, and oleic acids over linoleic acid distinguishes sesame oils from other eatable vegetable oils [63]. The ratios of saturated fatty acids to unsaturated fatty acids in the tested oil treatments ranged from 0.1620 to 0.1688, indicating a very high unsaturated fatty acid, which has implications from a nutritional standpoint.

### 3.8. Oxidizability, ODR, and LDR Values

The oxidizability (Cox value) as well as ODR and LDR indices in the current study showed no significant effects of the different roasting methods on the oil recovered from the sesame seeds. Table 4 shows that the microwave and local market roasting methods had the maximum and minimum Cox values, respectively. The Cox score for sesame oil is nearly constant, and it must be utilized to prevent vegetable oils from oxidative degeneration [64]. Other research stated that roasted sesame oils are more resistant to oxidation than raw sesame oils, and the significant oxidative constancy of sesame oils is due to the occurrence of lignin complexes and tocopherol [65,66]. Yen [57] indicated that roasted sesame oils have a higher oxidative steadiness due to the formation of antioxidant mixtures like tocopherol, sesamolin, and sesamol.

### 3.9. Chlorophyll and Carotenoid Contents According to Roasting Method

**Figure 1:**
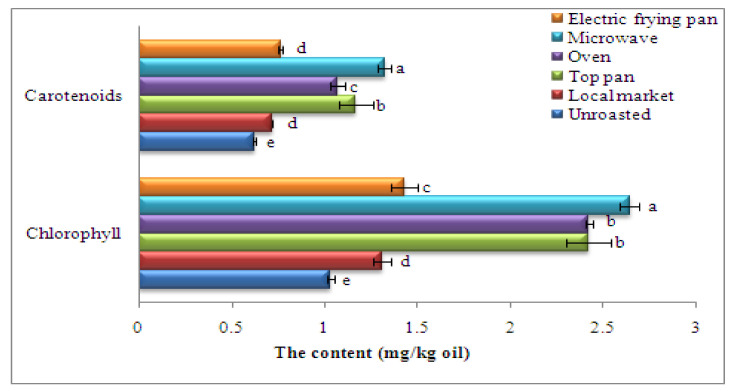
Chlorophyll and carotenoid contents (mg/kg oil) of oil recovered from sesame seeds roasted by different methods. Significant variations at *p* ≤ 0.05 are indicated by vertical bars with SD (n = 3) and different letters on the bars of every sample.

Natural oilseed carotenoid and chlorophyll are extracted into crude oil through industrial oil seed pressing or other recovery [67]. There were significant differences in chlorophyll and carotenoid contents affected by roasting methods (*p* ≤ 0.05) as given in Figure 1. The pre-treatment of sesame seed by different roasting methods before oil recovering showed the most oil recovered from seeds roasted in the microwave followed by stovetop pan and oven roasting (2.64, 2.42, and 2.42 mg/kg oil, respectively), in contrast with the unroasted seeds (1.03 mg/kg oil). This pattern was the same for carotenoid content. The current results correspond with Rekas et al. [68], who found gradual increases in the chlorophyll and carotenoid levels in rapeseed oil treated with microwave (800 W). Carotenoids are a wide range of pigments having bioactive functions that contribute to human health. Increased carotenoid levels may be because the breakdown between bound proteins and pigments enhances the liberation of carotenoids into the oil [44]; the pattern was seen for carotenoid content in flaxseed oil [69]. Carotenoids, different from chlorophylls, contribute both antioxidant properties and an attractive yellow colour to the oils [70]. The high carotenoid contents in the oil recovered from heat-treated seeds might be partially explained by the fact that carotenoids attach to proteins within seeds through thermal treatments or form heat-stable carotenoid–protein complexes. The oil’s affinityto lipid-soluble carotenoids is increased by thermal-induced protein denaturation or the degradation of interior seed cell structure [71]. The presence of oxygen, metals, enzymes, unsaturated lipids, prooxidants, and light all have a role in carotenoid oxidation [72].

### 3.10. Conjugated Dienes (CD) and Trienes (CT)

**Figure 2:**
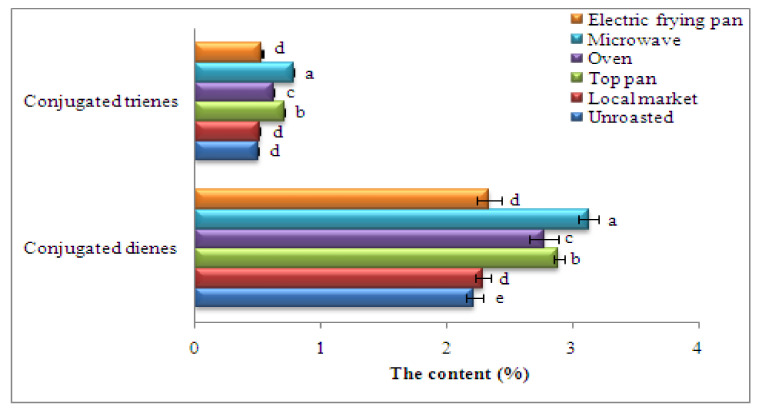
Conjugated diene and conjugated triene percentages in oils recovered from sesame seeds roasted by different methods. Significant variations at *p* ≤ 0.05 are indicated by vertical bars with SD (n = 3) and different letters on the bars of every sample.

The rates of conjugated dienes or conjugated trienes in unroasted sesame seeds oils were 2.220% and 0.502%, respectively. As clear from Figure 2, microwave roasting gave significantly more conjugated dienes and conjugated trienes than with other methods (3.128 and 0.788%, respectively; Figure 2). The figure shows that the conjugated diene values were higher than those of the conjugated trienes and this difference might be due to the conjugated trienes formed only on fatty acids containing three or more double bonds. Demnati et al. [73] suggested that an increase in the conjugated dienes and conjugated trienes in oil from roasted argan seed might be attributed to roasting at low temperature. Increasing conjugated diene or conjugated triene contents were associated with high prime lipid oxidation yields, and their reduction was associated with breakdowns of main lipid oxidation products throughout roasting. The amount of CD is significant for main oxidation, while the occurrence of CT designates the development of oxidation by products like unsaturated α-and β-diketones or β-ketones [67].

### 3.11. Tocopherol and Phytosterol Contents of Oils Recovered from Sesame Seeds Roasted by Different Methods

**Figure 3:**
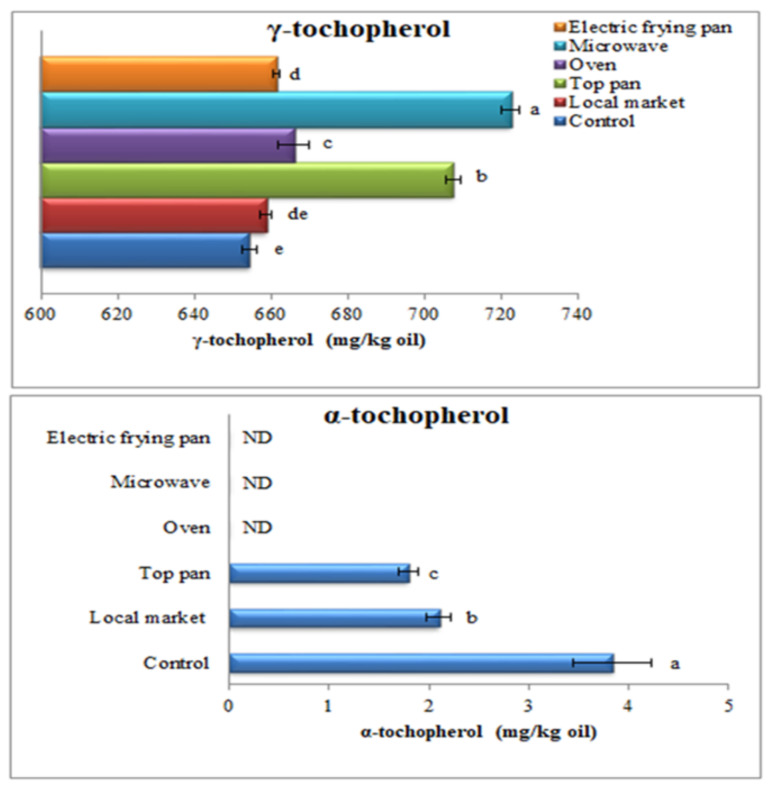
Tocopherol contents in oils recovered from sesame roasted with different methods. Significant variations at *p* ≤ 0.05 are indicated by vertical bars with SD (n = 3) and different letters on the bars of every sample.

Tocopherols have exceptional antioxidant activities for vegetable oils and have a critical function in fertility and illness avoidance. Tocopherols are crucial oil compounds because of their antioxidant properties, which may prevent unsaturated fatty acids from oxidation. Only α- and γ-tocopherol were found in the sesame oils used in the current investigation. Tocopherol’s patterns according to the different roasting methods are shown in Figure 3. It was observed that γ-tocopherol increased with roasting, and the maximum was obtained with microwave treatment (722.50 mg/kg oil) followed by stovetop pan (707.62 mg/kg oil). On the other hand, α-tochopherol was negatively affected by roasting; it was lower in the local market and stovetop pan-roasted samples (2.10 and 1.79 mg/kg oil, respectively) compared with the unroasted sample (3.84 mg/kg oil); however, there were no differences between oven, microwave, and electric frying pan roasting. Since 97% of the tocopherol in sesame oil is in the γ-form, only γ-tocopherol has been detected [57]. Phytosterol is an important factor for identifying corruptions or determining accuracy; it may be considered a fingerprint. Furthermore, because of their antioxidant action and health benefit, their identification is crucial [74]. Lee et al. [75] explained that the better release of vitamin E may be due to the destruction of the cell membrane through heating and the breaking of the covalent bonds between tocopherol and proteins or phospholipids, resulting in the releasing of more free tocopherol [76]. On the other hand, tocopherol content decreased with longer roasting times at higher temperatures due to cell breakdown and oxidative reaction [11]. These variations are likely due to differences in roasting methods, sesame seed genotypes or origins, post-harvest procedures, tocopherol recovery, and analysis condition [15]. Reductions of vitamin E content in oil were ascribed to cell damage and oxidative reaction through the roasting process [77]; hypothetically, the heat breaks down the bonds that link vitamin E to phospholipids and proteins [78].

**Figure 4:**
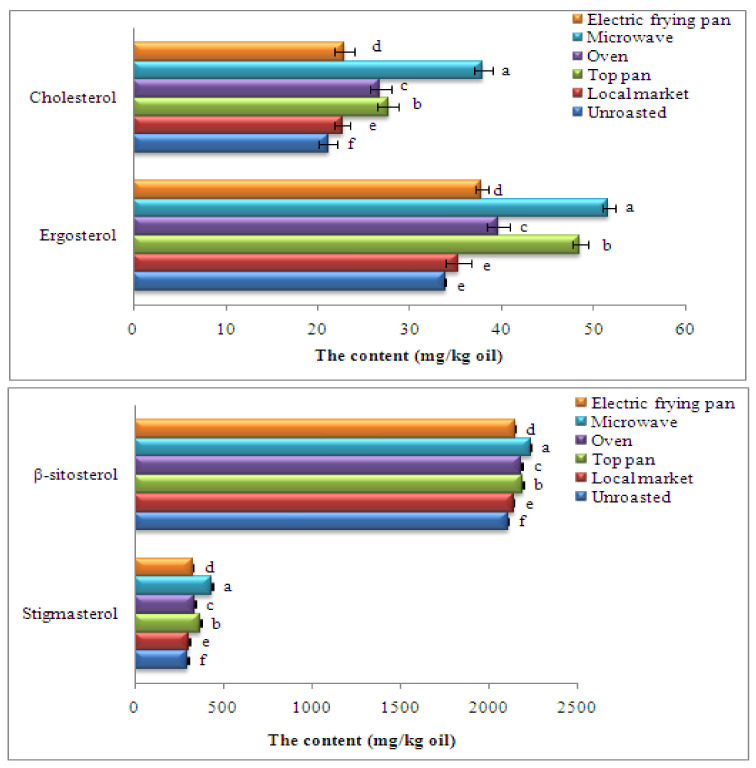
Phytosterol composition of oils recovered from sesame seeds roasted with different methods. Significant variations at *p* ≤ 0.05 are indicated by vertical bars with SD (n = 3) and different letters on the bars of every sample.

As is known, phytosterols are substances found in the unsaponifiable lipids portions of meals and are thought to provide health benefits. Phytosterols such as primarily β-sitosterol, campesterol, and stigmasterol are important natural compounds of plant cell membranes that are found in vegetable oils. Findings in Figure 4 show the phytosterol profiles of unroasted and roasted sesame oils by different methods. Four sterols were found, ergosterol, cholesterol, stigmasterol, and β-sitosterol, and stigmasterol and β-sitosterol were the most abundant phytosterols in the profiles; their contents were the highest with the microwave roasting (434.67 and 2235.12 mg/kg oil, respectively).

High phytosterol content is affected by the roasting type, possibly related to damage to the cell structure that improves phytosterol recovery. Wroniak et al. [79] detected structural alterations in proteins that harmed the lipoprotein membranes enclosing lipid bodies in microwave-treated rapeseed because heating encouraged denaturation. Gao et al. [80] indicated that roasting walnut kernels induced an important increase in phytosterol content in extracted oil compared with the oil recovered from the raw kernels. Lin et al. [81] found that total phytosterol contents of sunflower and rapeseed oil treated at 150 and 180 °C, respectively, for 16 min were higher than untreated oil. Clinical investigations have established that direct eating of phytosterol as a part of regular meals or as a complement helps to decrease cholesterol and inhibits numerous ailments as well as several malignancies [82,83,84].

### 3.12. Phenol, Flavonoid, and Antioxidant Activity of Oils Recovered from Sesame Seeds Roasted with Different Methods

**Figure 5:**
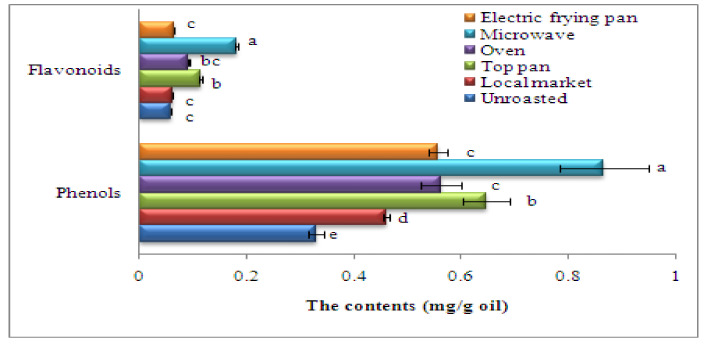
Phenol and flavonoid contents of oil recovered from sesame roasted with different methods. Significant variations at *p* ≤ 0.05 are indicated by vertical bars with SD (n = 3) and different letters on the bars of every sample.

**Figure 6:**
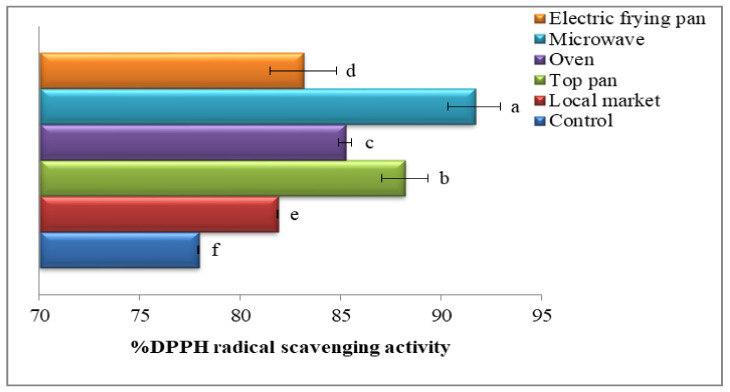
Antioxidant activity (%) of oils recovered from sesame roasted with different methods. Significant variations at *p* ≤ 0.05 are indicated by vertical bars with SD (n = 3) and different letters on the bars of every sample.

The phenol and flavonoid contents of the oils recovered from unroasted and roasted sesame seeds are given in Figure 5. The phenol and flavonoid contents were markedlyhigher in the roasted seeds than in the unroasted. The highest total phenolic and flavonoid contents were found for microwave roasting (0.8658 or 0.1812 mg/g oil, respectively). Furthermore, the antioxidant activity using DPPH radicals in unroasted as well as roasted sesame seed oils is shown in Figure 6. Roasting significantly increased the antioxidant activity (*p* ≤ 0.05), with the highest antioxidant activity recorded for microwave treatment (91.66%) followed by the stovetop pan roasting (88.19%). In short, phenol and flavonoid contents and antioxidant activity differ depending on the roasting method, and high antioxidant activity is attributed to high phenolic and flavonoid contents [85]. The increased phenols derived from the roasted sesame seed oils are most likely because of the heat breakdown of the cell walls and of the covalent bonds between phenolic compounds, which releases extra free phenols. Roasting, according to Jannat et al. [86], can enhance the concentrations of gamma-tocopherol and polyphenols in recovered sesame oils, resulting in developed radical scavenging capabilities. The synergistic impacts of the greater release of bioactive elements such as phytosterols and tocopherols, plus the formation of Maillard reaction products (MRPs) in the roasting process, liberate the antioxidant activities of roasted peanut oil [87]. Sesame oil has a higher oxidative constancy than other vegetables oil even though it includes about 85%USFAs. The occurrence of a large number of endogenous antioxidants such as phenols and tocopherols contributes to its extraordinary durability [88]. The enhancement in total antioxidant activities might be attributed to novel molecules formed during seed heating, like MRPs, which account for the enhanced constancy of the oil [89].

### 3.13. Thermogravimetric Analysis (TGA)

**Figure 7:**
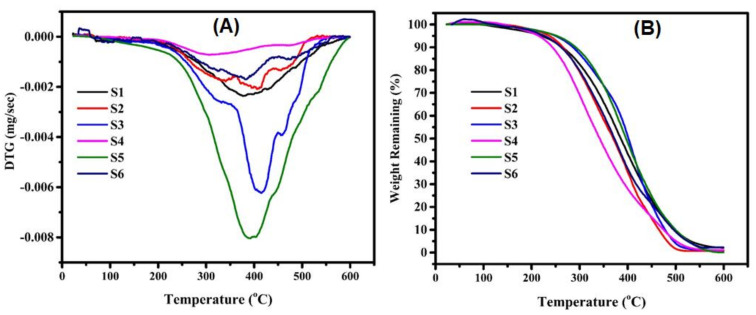
(**A**) Derivative thermogravimetric (DTG) and (**B**) thermogravimetric analysis (TGA) curves for oil recovered from sesame seeds roasted with different methods: S1: unroasted, S2: local market roasted, S3: stovetop pan, S4: oven, S5: microwave, and S6: electric frying pan.

The impact of oxidative conditions on the thermal constancy of the oil recovered since unroasted as well as roasted sesame seeds using different methods was studied by derivative thermogravimetric (DTG) and thermogravimetric analysis (TGA) as presented in Figure 7A,B. Degradation rate peaks are observed at 300, 360, 380, 390, 400, and 410 °C for stovetop pan, unroasted, oven, microwave, local market, and electric frying pan, respectively (Figure 7A). Generally, the unroasted and roasted oils contained three main hydrocarbon components, i.e., polyunsaturated, monounsaturated, and saturated fatty acids, all of which degrade during heating.

The DTG curves are similar for most oils, except for the one recovered the sesame seeds roasted by stovetop pan. The current findings are consistent with the fatty acid profiles obtained by GC, which showed that different roasting methods change the contents of the studied compounds and the structural characteristics of the roasted sesame seed oils. Figure 7B presents the thermal degradation of the oils recovered from sesame seeds roasted with different methods. The main degradation temperatures and weight loss were: 378 °C with 2%, 406 °C with 0.66%, 412 °C with 0.98%, 312 °C with 1.1%, 392 °C with 0.09, and 382 °C with 2.28 for unroasted, local market, stovetop pan, oven, microwave, and electric frying pan, respectively. The major decomposition stage indicates the breakdown of covalent bonds and modified chemical configurations, with the greatest weight loss about 400 °C. Tan et al. [90] reported that the oxidative stability of vegetable oils showed similar results and that the oils that contained high proportions of free fatty acids were more liable to oxidation; the authors determined that liberated FAs show less constant oxidation than FAs bound to glycerol molecules. Fatty acids with elongated carbon chains are more constant as a result of heating degradation than the short-chain FAs attributed to increasing intermolecular dispersal powers [91].

### 3.14. Fourier Transforms Infrared (FTIR) Spectroscopy

FTIR analysis provides a rapid means of evaluating the oxidative state of an oil or of monitoring changes in oil undergoing thermal stress [91].

**Figure 8:**
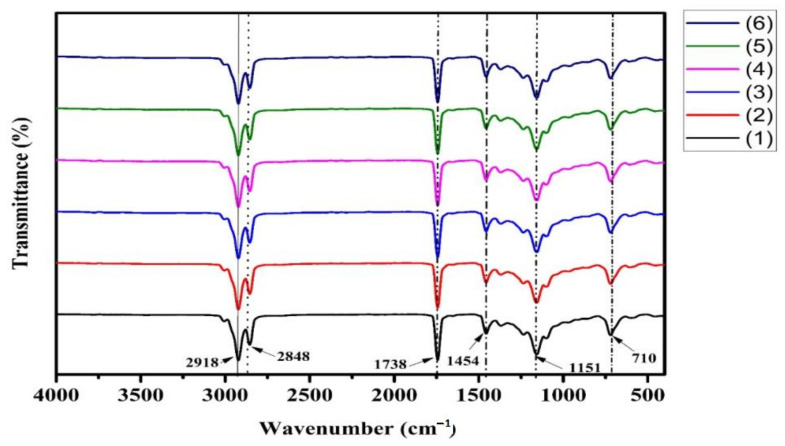
Spectral analysis obtained from FTIR for oils recovered from sesame seeds roasted with different methods: 1: Unroasted, 2: local market roasted, 3: stovetop pan, 4: oven, 5: microwave, and 6: electric frying pan.

The FTIR spectra of the sesame oils obtained from the selected roasting methods are presented in Figure 8, revealing similar band positions for all the roasting methods, which indicates similar components across methods. The main variation among the different roasting methods was the proportion transmittance (%T) of the same components. The assignments of functional groups responsible for IR absorption peak are as follows: 2918 and 2848 (symmetric and asymmetric stretching vibration, respectively, of CH_2_); 1738 (C = O stretching vibrations); 1454 (bending vibrations of the CH_2_ and CH_3_); 1151, (C–O stretching vibration), in accordance with the findings of Ali et al. [92] and Lerma-Garcia et al. [93]. The spectral changes appearing at the 2918 and 2848 cm^−1^ region after different roasting methods assist in monitoring oxidation and is related to the advanced state of oxidation of samples [94,95]. There was another major absorbance near 1738 cm^−1^ associated with the appearance of saturated aldehyde and carbonylic compound functional groups [96]. Similar findings were reported by Suri et al. [47], who obtained a sharp peak at 1745 cm^−1^ associated with the stretching–shaking of ester carbonyl functional sets of triglycerides. Valdés et al. [95] found a small peak at 1454 cm^−1^ on ATR–FTIR attributed to the bending–shaking of C-H bonds of CH_2_ aliphatic sets of triglycerides in almonds. Liang et al. [8] also found a sharp peak at 1151 cm^−1^ due to the stretching–shaking of C=O ester groups of triglycerides and of 1163 cm^−1^ for walnut oils during roasting. Finally, the authors found a peak at 710 cm^−1^ (associated by the out-of-plane vibration of cis‒HC = CH group of disubstituted olefines as well as overlapping of CH_2_ rocking shaking) [97,98].

The increased percent T in the esters could be associated with volatile compounds being liberated through the roasting of nuts, while the decreased percent T in first or second amines could be attributed to MRPs and the creation of colour or smell. In oil extracted from roasted seeds, all peaks were detected at allocated wave, and no movement of the peak was noted. According to the FTIR spectral investigation, the oils recovered from roasted seeds were stable and acceptable.

## 4. Conclusions

The current investigation focused on the properties of oil recovered from sesame seeds exposed to different roasting methods. Microwave roasting was the most favoured method because it is improved the contents of tocopherols and polyphenols as well asthe antioxidant activity and oxidative constancy of sesame seed oils and did not alter their structures. Phenols, flavonoids, and antioxidant activity were also highest with microwave roasting. Because roasted sesame seed oil contains a higher concentration of natural antioxidants like tocopherol and polyphenol, it is possible that roasting the seeds is a better pre-processing approach for boosting the nutritional value and health of sesame seed oils, specifically microwave roasting. The FTIR spectra indicated slight changes in peak intensity (1738, 1454, 1151, 710 cm^−1^) between the roasting methods used. It is suggested that the results of this research can be highly useful to guide further research on sesame seeds. The method of roasting affects the characteristics of sesame seed oils, and phytosterols were most abundant with microwave treatment. Using a microwave considerably improved the fat value in the seed.

## Figures and Tables

**Table 1:** Chemical properties of oil recovered from sesame seeds roasted by different methods.

Roasting Type	Lipid Content (%)	Peroxide Value (meq/kg Oil)	Iodine Value (g/100g Oil)	Acid Value (mg KOH/g Oil)	Saponification Value (mg KOH/g Oil)	Ester Value (mg/g Oil)
Unroasted	49.83 ± 0.11 e	1.59 ± 0.005 f	105.10 ± 1.01 a	1.03 ± 0.02 e	189.8 ± 0.31 d	188.8 ± 0.92 c
Local market	59.39 ± 0.03 b	1.93 ± 0.035 e	104.40 ± 0.47 c	2.65 ± 0.18 c	191.0 ± 0.78 cd	188.4 ± 0.71 c
Stovetop pan	50.65 ± 0.12 d	2.61 ± 0.050 d	104.70 ± 0.86 b	3.78 ± 0.05 a	193.2 ± 0.38 b	189.4 ± 0.88 c
Oven	58.74 ± 0.19 c	2.96 ± 0.025 c	102.90 ± 1.03 e	1.66 ± 0.02 d	195.3 ± 0.51 a	193.7 ± 0.79 a
Microwave	59.85 ± 0.18 a	3.90 ± 0.015 a	104.00 ± 1.04 d	3.24 ± 0.02 b	192.1 ± 1.28 bc	188.9 ± 1.28 c
Electric frying	59.75 ± 0.02 a	3.77 ± 0.040 b	102.30 ± 1.22 f	2.49 ± 0.15 c	194.7 ± 0.54 a	192.2 ± 0.63 b
LSD 0.05	0.26	0.05753	1.565	0.2074	1.419	1.332

Results are means ± SD, means in same column with various letters significantly differ (*p* ≤ 0.05).

**Table 2:** Fatty acid (%) profiles of oil recovered from unroasted and roasted sesame seeds with different roasting methods.

Fatty Acid	Sesame Roasting Methods					
	Unroasted	Local Market	Stovetop pan	Oven	Microwave	Electric Frying Pan
Palmitic acid C16:0	8.09 ± 0.01 b	8.16 ± 0.02 b	8.15 ± 0.01 b	8.53 ± 0.03 a	8.12 ± 0.01 b	8.13 ± 0.02 b
Palmitoleic C16:1	0.66 ± 0.02 b	0.60 ± 0.01 c	0.61 ± 0.01 c	0.57 ± 0.02 c	0.77 ± 0.03 a	0.53 ± 0.02 c
Stearic C18:0	5.51 ± 0.03 c	5.53 ± 0.01 c	5.75 ± 0.04 a	5.63 ± 0.02 b	5.65 ± 0.03 b	5.60 ± 0.02 b
Oleic C18:1	41.28 ± 0.06 a	41.24 ± 0.11 a	41.38 ± 0.09 a	41.53 ± 0.12 a	41.48 ± 0.1 a	41.33 ± 0.08 a
Linoleic C 18:2	43.27 ± 0.03 d	43.17 ± 0.02 e	43.68 ± 0.04 b	43.63 ± 0.03 b	43.84 ± 0.06 a	43.55 ± 0.05 c
Linolenic acid C18:3	0.55 ± 0.01 b	0.50 ± 0.02 c	0.60 ± 0.01 a	0.55 ± 0.02 b	0.60 ± 0.02 a	0.55 ± 0.01 b
Arachidic C20:0	0.29 ± 0.01 c	0.35 ± 0.01 a	0.32 ± 0.01 b	0.40 ± 0.02 a	0.39 ± 0.01 a	0.37 ± 0.02 a
ΣSFAs	13.89 ± 0.03 f	14.04 ± 0.01 e	14.22 ± 0.03 b	14.56 ± 0.04 a	14.16 ± 0.02 c	14.10 ± 0.02 d
ΣMUFAs	41.94 ± 0.03 c	41.84 ± 0.03 d	41.99 ± 0.03 c	42.10 ± 0.04 b	42.25 ± 0.04 a	41.86 ± 0.02 d
ΣPUFAs	43.82 ± 0.02 e	43.67 ± 0.04 f	44.28 ± 0.04 b	44.18 ± 0.03 c	44.44 ± 0.05 a	44.10 ± 0.03 d
ΣUFAs	85.76 ± 0.03 d	85.51 ± 0.03 e	86.27 ± 0.04 b	86.28 ± 0.03 b	86.69 ± 0.04 a	85.96 ± 0.02 c
SFA/UFAs	0.1620 ± 0.02 b	0.1642 ± 0.02 b	0.1648 ± 0.01 b	0.1688 ± 0.01 a	0.1633 ± 0.01 b	0.1640 ± 0.02b

Results are means ± SD, means in same row with various letters are significantly different (*p* ≤ 0.05). SFAs: Saturated fatty acids; MUFAs: mono-unsaturated fatty acids; PUFAs: Polyunsaturated fatty acids UFAs: unsaturated fatty acids.

**Table 3:** Ratios of palmitic, stearic, and oleic acids over linoleic acid and SFA/USFA ratios of oil recovered from sesame seeds roasted with different methods.

Roasting Type	P/L	S/L	O/L	SFAs/USFAs
Unroasted	0.1870	0.1273	0.9540	0.1620
Local market	0.1890	0.1281	0.9553	0.1642
Stovetop pan	0.1866	0.1316	0.9473	0.1648
Oven	0.1955	0.1290	0.9519	0.1688
Microwave	0.1852	0.1289	0.9462	0.1633
Electric frying pan	0.1867	0.1286	0.9490	0.1640

**Table 4:** Oxidizability, ODR, and LDR indices for oil recovered from sesame seeds roasted by different methods.

Roasting Type	Oxidizability Value	Oleic Desaturation Ratio (ODR)	Linoleic Desaturation Ratio (LDR)
Unroasted	4.99	0.5149	0.0126
Local market	4.97	0.5143	0.0115
Stovetop pan	5.04	0.5169	0.0136
Oven	5.03	0.5155	0.0124
Microwave	5.06	0.5172	0.0135
Electric frying pan	5.02	0.5162	0.0125

## Data Availability

Data are contained within the article.
